# Plasma biomarkers for predicting the development of dementia in a community‐dwelling older Japanese population

**DOI:** 10.1111/pcn.13661

**Published:** 2024-04-12

**Authors:** Tomoyuki Ohara, Harutsugu Tatebe, Jun Hata, Takanori Honda, Mao Shibata, Sayo Matsuura, Tatsuya Mikami, Tetsuya Maeda, Kenjiro Ono, Masaru Mimura, Kenji Nakashima, Jun‐ichi Iga, Minoru Takebayashi, Takahiko Tokuda, Toshiharu Ninomiya, JPSC‐AD (Japan Prospective Studies Collaboration for Aging and Dementia) study group

**Affiliations:** ^1^ Department of Neuropsychiatry, Graduate School of Medical Sciences Kyushu University Fukuoka Japan; ^2^ Department of Epidemiology and Public Health, Graduate School of Medical Sciences Kyushu University Fukuoka Japan; ^3^ Department of Functional Brain Imaging Institute for Quantum Medical Science Chiba Japan; ^4^ National Institutes for Quantum Science and Technology Chiba Japan; ^5^ Department of Medicine and Clinical Science, Graduate School of Medical Sciences Kyushu University Fukuoka Japan; ^6^ Center for Cohort Studies, Graduate School of Medical Sciences Kyushu University Fukuoka Japan; ^7^ Department of Psychosomatic Medicine, Graduate School of Medical Sciences Kyushu University Fukuoka Japan; ^8^ Department of Preemptive Medicine, Graduate School of Medicine Hirosaki University Hirosaki Japan; ^9^ Division of Neurology and Gerontology, Department of Internal Medicine, School of Medicine Iwate Medical University Iwate Japan; ^10^ Department of Neurology Kanazawa University Graduate School of Medical Sciences, Kanazawa University Kanazawa Japan; ^11^ Department of Neuropsychiatry Keio University School of Medicine Tokyo Japan; ^12^ National Hospital Organization Matsue Medical Center Shimane Japan; ^13^ Department of Neuropsychiatry Ehime University Graduate School of Medicine, Ehime University Ehime Japan; ^14^ Faculty of Life Sciences, Department of Neuropsychiatry Kumamoto University Kumamoto Japan

**Keywords:** Alzheimer disease, dementia, plasma biomarker, prediction, prospective study

## Abstract

**Aim:**

To assess the association between plasma amyloid β (Aβ) 42/40, phosphorylated tau (p‐τ)181, glial fibrillary acidic protein (GFAP), or neurofilament light chain (NfL) and the risk of dementia and to determine whether these plasma biomarkers could improve the ability to predict incident dementia in a general older population.

**Methods:**

A total of 1346 Japanese community‐dwelling individuals aged ≥65 years without dementia were followed prospectively for 5.0 years. Plasma biomarkers were quantified using a Simoa HD‐X analyzer. A Cox proportional hazards model was used to estimate the hazard ratios of each plasma biomarker level for the risk of dementia.

**Results:**

During the follow‐up, 151 participants developed dementia, of whom 108 had Alzheimer disease (AD) and 43 non–Alzheimer dementia (non‐AD). Lower plasma Aβ42/40 levels and higher plasma p‐τ181 levels were significantly associated with developing AD but not non‐AD, whereas significant associations were observed between higher plasma levels of GFAP and NfL and risk of both AD and non‐AD (all *P* for trend <0.05). In addition, adding these four plasma biomarkers into a model consisting of the total score of the dementia risk model significantly improved the predictive ability for incident dementia.

**Conclusion:**

Our findings suggest that plasma Aβ42/40 and p‐τ181 are specific markers of AD, and plasma GFAP and NfL are potential biomarkers for all‐cause dementia in the general Japanese older population. In addition, the measurement of these plasma biomarkers may be a useful and relatively low‐invasive procedure for identifying individuals at high risk for developing dementia in clinical practice.

Lecanemab was recently given full approval from the US Food and Drug Administration as a disease‐modifying drug for Alzheimer disease (AD),^1^ creating an urgent need for the identification of minimally invasive, low‐cost, and time‐effective tools for screening and risk stratification of patients with any level of cognitive decline. Established biomarkers for the diagnosis of AD include amyloid β (Aβ) and phosphorylated tau (p‐τ) in cerebrospinal fluid (CSF) and on positron emission tomography (PET).^2^ However, because measurement of these biomarkers in the CSF or on PET is invasive and expensive, it is not widely used in primary care and clinical office‐based settings.^3^ Blood‐based biomarkers have thus garnered much attention as an alternative, first‐line screening method that is readily available and relatively noninvasive in clinical practice.

Recently, Teunissen et al. provided a comprehensive overview of four plasma biomarkers: plasma Aβ, p‐τ, glial fibrillary acidic protein (GFAP), and neurofilament light chain (NfL).^4^ Aβ is the main pathological marker of AD, and some prospective studies have reported that a low plasma Aβ42/40 ratio (Aβ42/40) is significantly associated with risk of developing AD.^5^, ^6^, ^7^, ^8^, ^9^, ^10^ p‐τ is another pathological marker of AD,^2^ and clinical and neuropathological studies have shown that plasma p‐τ181 may be an AD‐specific neuropathological biomarker of the disease burden in the early stages, increasing at least 8 years before neuropathological confirmation of AD.^9^, ^11^, ^12^ Immunological processes play a major role in the development of dementia, and GFAP is known as a marker of astrocyte reactivity or astrocytosis that is commonly found surrounding Aβ plaques.^13^ Clinical studies have reported that plasma GFAP is an early marker of brain Aβ accumulation, and increased plasma GFAP levels secondary to Aβ aggregation may promote p‐τ accumulation.^4^, ^14^ Only two prospective studies have assessed the association between serum GFAP levels and risk of dementia,^15^, ^16^ and no prospective studies, to our knowledge, have investigated this association using plasma GFAP. NfL is known as a robust marker of neurodegeneration irrespective of its pathological cause^4^; accordingly, a few prospective studies have assessed the association between plasma or serum NfL levels and risk of dementia.^8^, ^16^, ^17^ However, to our knowledge, there have been no population‐based prospective longitudinal studies that have comprehensively assessed the association between plasma levels of Aβ42/40, p‐τ181, GFAP, and NfL and the risk of developing dementia.

The objectives of this study were to elucidate the association between plasma levels of Aβ42/40, p‐τ181, GFAP, and NfL and the risk of developing dementia and to clarify whether these four plasma biomarkers would improve the ability to predict incident dementia in a general Japanese older population.

## Materials and Methods

### Study population

This study was conducted as a substudy of the JPSC‐AD (Japan Prospective Studies Collaboration for Aging and Dementia) using longitudinal data from the Hisayama cohort.^18^ In the Hisayama cohort, which is an ongoing population‐based prospective cohort study of cardiovascular diseases and dementia, repeated full‐community surveys for dementia have been conducted every 5 to 7 years since 1985 in the town of Hisayama, a suburb of Fukuoka City in southern Japan.^19^ Among 2036 town residents aged 65 years and older in 2012–2013, a total of 1906 residents (1126 women and 780 men) (participation rate: 93.6%) participated in the examination for cognitive function and health status. After excluding 44 participants who did not consent to participate in the study, 339 participants who had dementia at baseline, 174 participants without available plasma, two participants who did not complete the examination for cognitive function at baseline, and one participant with intellectual disability, the remaining 1346 participants (765 women and 581 men) were enrolled in the present study. The present study was conducted with the provisions of the Declaration of Helsinki and the approval of the Kyushu University and National Institutes for Quantum Science and Technology Institutional Board of Clinical Research (approval numbers 686–10, 2023–56, and 2022–24, respectively). We obtained written informed consent from all the participants.

### Follow‐up surveys

The participants were followed for a median of 5 years (interquartile range [IQR], 4.9–5.1 years) from the baseline examination. As reported previously,^19^, ^20^ we used an established daily monitoring system comprising the study team, local physicians, and members of the town's Health Office to regularly collect information on new neurologic events, including any cognitive decline and stroke. We also conducted regular health examinations annually to identify incident cases of dementia. Postal and telephone surveys were performed for participants who did not undergo regular health examinations or moved out of town. Moreover, to precisely detect dementia cases to the greatest extent possible, we conducted comprehensive neuropsychological screening for dementia in 2017–2018,^21^ with 1159 patients participating (86.1% of total participants). When dementia or any neurological symptoms including cognitive impairment were suspected, a psychiatrist and stroke physician from the study team carefully evaluated the participant for the presence or absence of dementia. In addition, when a participant died, we conducted comprehensive investigations, including interviews of the family or attending physician and a review of all the available clinical records, including neuroimaging (computed tomography/magnetic resonance imaging). The participants were followed up until the date of neuropsychological screening for dementia in 2017–2018 or March 31, 2018, for those who did not participate in the neuropsychological screening in 2017–2018. No participants were lost to follow‐up except for deceased cases.

### Diagnosis of dementia

The diagnoses of dementia and mild cognitive impairment (MCI) were made using the criteria of the DSM‐III, Revised,^22^ and the clinical criteria reported by Petersen *et al*. in 2001,^23^ respectively. Subtypes of dementia were diagnosed as AD or non–Alzheimer dementia (non‐AD) based on the diagnostic criteria of the National Institute of Neurological and Communicative Disorders and Stroke and the Alzheimer's Disease and Related Disorders Association.^24^ In the screening survey, we used the Mini‐Mental State Examination (MMSE).^25^, ^26^ For participants who were suspected of having dementia or MCI, expert psychiatrists conducted comprehensive cognitive and neurological evaluations including the Wechsler Memory Scale of logical memory.^27^, ^28^ We defined MCI as either of: (i) objective cognitive impairment based on the neuropsychological data; or (ii) any cognitive complaint by a family member, the town's Health Office members, or local practitioners in individuals who showed no evidence of dementia. Every case of dementia and MCI was adjudicated by expert psychiatrists and stroke physicians on the study team.

### Measurement of plasma biomarkers

In 2012–2013, we collected plasma samples as part of the survey. Ethylenediaminetetraacetic acid blood samples were centrifuged at 1500*g* for 10 min after allowing blood to clot at room temperature for 30 min. Separated plasma was collected into polypropylene tubes and frozen at −80°C within 3.5 to 6.0 h after collection. In 2023, we thawed these plasma samples and quantified plasma levels of the A/T/N biomarkers (Aβ42, Aβ40, p‐τ181, and NfL) and GFAP utilizing a SimoaHD‐X analyzer (Quanterix) with a Simoa Human Neurology 4‐Plex E kit and Simoa pTau‐181 V2 Advantage kit (Quanterix) according to the manufacturer's instructions. We used Aβ42/40 as a proxy for the cerebral amyloid burden in accordance with previous studies.^4^, ^29^ All plasma samples were run in duplicate with the same lot of standards. The relative concentration estimates of plasma biomarkers were calculated according to the respective standard curves. Aβ42/40 data were not available for one participant.

### Risk factor measurements

In the baseline survey, a self‐administered questionnaire on lifestyle factors including educational status, smoking habits, alcohol intake, medical history, and treatment of diabetes, hypertension, and hypercholesterolemia was administered by trained interviewers. We defined low education as ≤9 formal educational years. Blood pressure was measured three times after more than 5 min of rest in the sitting position and the mean value of the three measurements was calculated. Hypertension was defined as current use of antihypertensive agents and/or blood pressure ≥ 140/90 mm Hg. We measured plasma glucose levels by using the hexokinase method and determined diabetes as follows: fasting glucose level ≥7.0 mmol/L, casual or 2‐h 75‐g oral glucose postloaded glucose level ≥ 11.1 mmol/L, and/or use of insulin or oral hypoglycemic agents.^30^ We also measured serum total cholesterol levels enzymatically and defined hypercholesterolemia as serum cholesterol ≥5.69 mmol/L and/or use of lipid‐lowering agents. Serum creatinine concentrations were measured using an enzymatic method. Estimated glomerular filtration rate (eGFR) was calculated using the Chronic Kidney Disease Epidemiology Collaboration equation with a Japanese coefficient of 0.813.^31^ eGFR was also classified into three groups according to the KDIGO 2021 guideline^32^ as follows: ≥60, 30 to 59, and <30 mL/min/1.73 m^2^. History of stroke and history of cerebrocardiovascular disease were determined by using all clinical information of the Hisayama Study. We measured body height and weight in light clothing without shoes and calculated body mass index (BMI; kg/m^2^). Electrocardiogram abnormalities were defined as Minnesota Code 3–1, 4–1, 4–2, 4–3, or 8–3. We classified alcohol intake and smoking habits as being either current habitual or not. Regular exercise was defined as engaging in sports or other forms of exercise at least three times a week during leisure time. Daily physical activity levels were reported for common occupational/domestic activities as follows: mostly sitting or lying down all day, mixed sitting, standing and walking, walking, and heavy labor. Responses were classified as sedentary (i.e. mostly sitting or lying down) or not. To determine the APOE‐ε4 carrier, two single nucleotide polymorphisms (rs429358 and rs7412) were genotyped using the multiplex polymerase chain reaction–based Invader assay^33^ or the multiplex polymerase chain reaction–based targeted sequencing method^34^ as previously reported.

### Statistical analysis

We assessed correlations for each plasma biomarker level or between each log‐transformed plasma biomarker level and age, sex, and the MMSE by using Spearman correlation coefficient. The age‐ and sex‐adjusted mean values or frequencies of risk factors across the quartiles of each plasma biomarker level were computed by using a linear or logistic regression analysis, respectively. A Cox proportional hazards model was used to estimate the hazard ratios (HRs) with their 95% confidence intervals (CIs) for the incidence of dementia for quartiles of each plasma biomarker level. In the multivariable‐adjusted model, age, sex, low education, hypertension, diabetes, BMI, history of stroke, smoking habits, and sedentariness were included as covariates. These covariates were the same predictors included in our previously developed clinical prediction model for the development of dementia.^35^ In addition, we conducted another multivariate‐adjusted analysis adding eGFR and APOE‐ε4 carrier to these covariates. To assess the shape of the association between each plasma biomarker level and the risk of dementia and its subtypes, we used a restricted cubic spline analysis with four knots placed at the fifth, 35th, 65th, and 95th percentiles of each plasma biomarker level (0.03995, 0.06060, 0.07016, and 0.08596 for Aβ42/40; 2.298, 3.572, 4.845, and 9.099 pg/mL for p‐τ181; 85.531, 140.835, 197.308, and 376.407 pg/mL for GFAP; and 15.114, 24.159, 34.191, and 77.594 pg/mL for NfL, respectively).^36^ The fifth percentile of each plasma biomarker was set as the reference value. We tested for nonlinearity based on the likelihood ratio test by comparing the log‐likelihood of the model containing the linear term with that of the model containing cubic spline terms.^36^, ^37^ In addition, the risk‐predictive performance of the plasma biomarkers of interest for incident dementia were assessed by adding each plasma biomarker to the predicted dementia risk calculated by the aforementioned clinical prediction model,^35^ where the DeLong method was used to assess the consistency in the Harrell concordance statistics (C statistics) among models.^38^ The cutoff value for each plasma biomarker in association with the risk of dementia was evaluated using receiver operating characteristic curves; the cutoff value was taken as the point on the curve that comes closest to the (0.1) coordinate. We also examined the increased predictive ability of each plasma biomarker by using net reclassification improvement (NRI) and integrated discrimination improvement (IDI),^39^ where the individual probabilities were estimated by using the Cox proportional hazards model. The software package SAS version 9.4 (SAS Institute Inc) was used to perform all statistical analyses, and statistical significance was set at a two‐tailed *P* value of <0.05 in all analyses.

## Results

The median values of plasma concentrations for Aβ42/40, p‐τ181, GFAP, and NfL in this population were 0.0652 (IQR, 0.0569–0.0736), 4.12 pg/mL (IQR, 3.199–4.466 pg/mL), 165.625 pg/mL (IQR, 126.539–227.047 pg/mL), and 28.781 pg/mL (IQR, 21.682–39.272 pg/mL), respectively. Correlation coefficients between each plasma biomarker and the correlation plots between each log‐transformed plasma biomarker and age, sex, and the MMSE are shown in Table S1 and Figures S1 and S2, respectively. Tables 1 and 2 show the age‐ and sex‐adjusted clinical characteristics of the study population according to the quartiles of each plasma measure. For Aβ42/40, compared with participants with the highest quartile, the frequencies of women and alcohol intake were significantly lower and the mean values of systolic blood pressure and eGFR were marginally/significantly lower in those with the lowest quartile, while the frequency of diabetes was significantly higher in those with the lowest quartile. For p‐τ181, participants with the highest quartile had a significantly higher mean value of age, a marginally higher frequency of diabetes, and a significantly lower mean value of eGFR and frequencies of women and alcohol intake than those with the lowest quartile. For GFAP, compared with participants with the lowest quartile, those with the highest quartile had a significantly higher mean value of age and frequencies of women, history of stroke, and history of cerebrocardiovascular disease, and significantly lower mean values of systolic blood pressure, eGFR, and BMI and frequencies of antihypertensive agent use, hypertension, diabetes, smoking habits, and alcohol intake. For NfL, participants with the highest quartile had significantly higher mean values of age and frequencies of use of antihypertensive agents, diabetes, history of stroke, history of cerebrocardiovascular disease, and sedentariness, and significantly lower mean values of diastolic blood pressure, total cholesterol, eGFR, BMI, and frequencies of women and alcohol intake than those with the lowest quartile.

**Table 1:** Age‐ and sex‐adjusted baseline characteristics of participants according to plasma quartile levels of Aβ42/40 and p‐τ181: 2012–2013

	Aβ42/40				P‐τ181 (pg/mL)			
	Q1	Q2	Q3	Q4	Q1	Q2	Q3	Q4
Variables	*n* = 336	*n* = 336	*n* = 337	*n* = 336	*n* = 336	*n* = 337	*n* = 337	*n* = 336
Age, mean (SE), years	75 (0.36)	74 (0.36)	73 (0.36)†	74 (0.36)	72 (0.34)	73 (0.34)	75 (0.34)*	77 (0.34)*
Women, %	49.3*	57.1	58.8	62.2	63.7	62.4	55.3	45.9*
Education ≤9 years, %	37.1	35.1	41.0	36.2	41.6	36.8	36.3	34.9
Systolic BP, mean (SE), mm Hg	133 (1.02)*	135 (1.01)	135 (1.01)	136 (1.02)	136 (1.03)	134 (1.02)	134 (1.02)	136 (1.04)
Diastolic BP, mean (SE), mm Hg	76 (0.59)	77 (0.59)	76 (0.59)	77 (0.59)	78 (0.60)	76 (0.60)*	76 (0.60)*	77 (0.61)
Antihypertensive agent, %	53.4	61.2	54.4	55.8	54.0	52.1	58.2	60.4
Hypertension, %	70.6	71.5	74.8	72.9	70.6	71.5	74.8	72.9
Diabetes, %	26.4*	24.6	24.1	19.7	20.9	20.9	27.3†	25.7†
Serum total cholesterol, mean (SE), mmol/L	5.09 (0.05)	5.17 (0.05)	5.03 (0.05)	5.07 (0.05)	5.11 (0.05)	5.12 (0.05)	5.08 (0.05)	5.05 (0.05)
Serum HDL cholesterol, mean (SE), mmol/L	1.64 (0.02)	1.64 (0.02)	1.65 (0.02)	1.68 (0.02)	1.62 (0.02)	1.64 (0.02)	1.65 (0.02)	1.69 (0.02)
Hypercholesterolemia, %	54.0	54.4	54.7	56.7	57.0	54.9	55.2	52.8
eGFR (mL/min/1.73 m2), mean (SE)	64.4*	63.6	63.8	62.3	66.8	65.1*	63.4*	58.8*
Body mass index, mean (SE), kg/m^2^	22.9 (0.18)	23.3 (0.18)*	23.6 (0.18)*	22.6 (0.18)	23.1 (0.19)	23.0 (0.19)	23.3 (0.18)	23.1 (0.19)
Electrocardiogram abnormalities, %	15.6	16.8	16.2	16.0	17.8	11.6	16.3	18.9
Atrial fibrillation, %	1.7	3.5	2.4	3.9	2.8	1.2	3.2	4.4
History of stroke, %	3.8	4.6	7.0	5.3	4.9	5.4	5.3	5.1
History of cerebrocardiovascular disease	6.1	7.1	10.2	8.5	8.9	7.0	7.2	8.8
Smoking habits, %	5.1	5.7	5.4	6.9	6.6	5.8	5.8	4.9
Alcohol intake, %	40.8†	39.7	37.0	34.9	39.3	41.2	40.0	32.3†
Sedentariness, %	1.9	2.3	1.9	4.4	1.6	1.9	3.5†	3.3
Regular exercise, %	20.2	17.7	19.3	17.9	20.5	18.6	19.1	16.6
MMSE, mean (SE)	28 (0.15)	27 (0.14)	27 (0.14)	28 (0.14)	27 (0.15)	28 (0.14)	27 (0.15)	27 (0.15)
APOE‐ε4 carrier, %	25.2*	20.8*	13.8	11.5	11.3	12.4	20.9*	27.1*

All values are given as the mean or as a percentage adjusted for age and sex. Mean age was sex‐adjusted. Percentage of women was age‐adjusted. Electrocardiogram abnormalities were defined as Minnesota Code 3–1, 4–1, 4–2, 4–3, or 8–3. Hypercholesterolemia was defined as serum total cholesterol ≥5.69 mmol/L or use of lipid‐lowering drugs. Regular exercise was defined as engaging in sports at least three times per week during leisure time. *P < 0.05 vs quartile 4 (Q4); ^†^ *P* < 0.10 vs Q4 for amyloid β42/40 ratio (Aβ42/40), *P < 0.05 vs quartile 1 (Q1); †*P* < 0.10 vs Q1 for phosphorylated tau (p‐τ)181.

BP, blood pressure; eGFR, estimated glomerular filtration rate; HDL, high‐density lipoprotein; MMSE, Mini‐Mental State Examination; Q2, quartile 2; Q3, quartile 3; SE, standard error.

**Table 2:** Age‐ and sex‐adjusted baseline characteristics of participants according to plasma quartile levels of GFAP and NfL: 2012–2013

	GFAP (pg/mL)				NfL (pg/mL)			
	Q1	Q2	Q3	Q4	Q1	Q2	Q3	Q4
Variables	*n* = 336	*n* = 337	*n* = 337	*n* = 336	*n* = 336	*n* = 337	*n* = 337	*n* = 336
Age, mean (SE), years	70 (0.32)	72 (0.32)*	75 (0.32)*	79 (0.32)*	70 (0.30)	72 (0.30)*	75 (0.30)*	80 (0.30)*
Women, %	46.9	54.1	59.5†	66.8*	63.5	58.9	55.6*	49.3*
Education ≤9 years, %	39.7	36.7	38.1	34.9	41.3	34.0	37.0	37.2
Systolic BP, mean (SE), mm Hg	138 (1.06)	134 (1.02)*	134 (1.01)*	133 (1.09)*	137 (1.09)	134 (1.02)†	134 (1.02)†	135 (1.13)
Diastolic BP, mean (SE), mm Hg	77 (0.62)	76 (0.60)	76 (0.59)	76 (0.64)	78 (0.64)	76 (0.59)*	76 (0.59)*	76 (0.66)*
Antihypertensive agent, %	63.0	53.7*	54.2*	53.8*	52.7	53.2	54.7	64.2*
Hypertension, %	78.5	71.4*	70.5*	69.4*	72.2	70.2	70.0	77.5
Diabetes, %	28.6	22.1*	22.8†	21.3*	18.3	19.6	26.0*	31.3*
Serum total cholesterol, mean (SE), mmol/L	5.12 (0.05)	5.08 (0.05)	5.11 (0.05)	5.05 (0.05)	5.18 (0.05)	5.12 (0.05)	5.12 (0.05)	4.95 (0.05)*
Serum HDL cholesterol, mean (SE), mmol/L	1.63 (0.02)	1.66 (0.02)	1.64 (0.02)	1.69 (0.02)	1.61 (0.02)	1.68 (0.02)*	1.69 (0.02)*	1.64 (0.03)
Hypercholesterolemia, %	56.0	53.1	57.8	52.9	57.2	54.6	53.4	54.7
eGFR (mL/min/1.73 m2), mean (SE)	67.7	65.4*	62.0*	58.9*	68.9	66.6*	63.6*	55.0*
Body mass index, mean (SE), kg/m^2^	24.1 (0.19)	23.2 (0.18)*	22.8 (0.18)*	22.3 (0.19)*	23.8 (0.20)	23.2 (0.19)*	23.0 (0.18)*	22.6 (0.20)*
Electrocardiogram abnormalities, %	15.1	14.2	17.1	18.2	15.4	19.1	11.7	18.4
Atrial fibrillation, %	2.4	3.3	2.9	2.9	2.1	4.0	2.1	3.2
History of stroke, %	2.8	4.4	5.9†	8.0*	2.1	2.4	5.6*	12.1*
History of cerebrocardiovascular disease	5.6	5.8	8.5	12.3*	4.4	4.6	7.3	16.9*
Smoking habits, %	7.4	6.6	5.5	3.8†	7.2	5.3	3.8*	7.1
Alcohol intake, %	42.4	43.2	33.7†	33.6†	42.4	41.0	38.3	31.2*
Sedentariness, %	1.5	3.1	2.8	3.0	0.4	1.8	2.0	6.5*
Regular exercise, %	17.9	18.9	17.8	20.4	17.6	20.7	20.6	16.1
MMSE, mean (SE)	27 (0.15)	28 (0.15)	27 (0.15)	27 (0.15)	27 (0.16)	28 (0.15)	28 (0.14)	27 (0.16)
APOE‐ε4 carrier, %	12.9	16.5	19.6*	22.8*	16.7	17.1	19.0	18.7

All values are given as the mean or as a percentage adjusted for age and sex. Mean age was sex‐adjusted. Percentage of women was age‐adjusted. Electrocardiogram abnormalities were defined as Minnesota Code 3–1, 4–1, 4–2, 4–3, or 8–3. Hypercholesterolemia was defined as serum total cholesterol ≥5.69 mmol/L or use of lipid‐lowering drugs. Regular exercise was defined as engaging in sports at least three times per week during leisure time.

BP, blood pressure; eGFR, estimated glomerular filtration rate; GFAP, glial fibrillary acid protein; HDL, high‐density lipoprotein; MMSE, Mini‐Mental State Examination; NfL, neurofilament light chain; Q2, quartile 2; Q3, quartile 3; Q4, quartile 4; SE, standard error.

* *P* < 0.05 vs quartile 1 (Q1).

^†^ *P* < 0.10 vs Q1.

During the median follow‐up period of 5 years (IQR, 4.9–5.1 years), 151 participants (60 men and 91 women) developed all‐cause dementia. Of these, 143 participants with dementia were evaluated by brain imaging. Among them, 17 participants with dementia also underwent a brain autopsy. Regarding the subtypes of dementia, 108 participants were diagnosed as having AD, including mixed‐type AD (e.g. AD and vascular dementia), and 43 cases were counted as an event in the analysis for non‐AD.

Table 3 shows the estimated risks of all‐cause dementia according to the levels of each plasma biomarker. The age‐ and sex‐adjusted risk of all‐cause dementia increased significantly with lower plasma Aβ42/40 levels and higher GFAP and NfL levels (all *P* for trend <0.001), but no significant association was observed for plasma p‐τ181 levels. These associations remained significant after adjustment for age, sex, low education, hypertension, diabetes, eGFR, BMI, history of stroke, smoking habits, sedentariness, and APOE‐ε4 (all *P* for trend <0.001), except in the case of plasma p‐τ181.

**Table 3:** Association of plasma quartile levels of each biomarker with the risk of developing all‐cause dementia: 2012–2017

			Hazard ratio (95% CI)		
Plasma biomarker levels	Persons at risk	No. of events	Age‐ and sex‐adjusted	Multivariable‐adjusted^†^	Multivariable‐adjusted^‡^
**Aβ42/40**					
Q4 (0.0736–0.2172)	336	28	1.00 (reference)	1.00 (reference)	1.00 (reference)
Q3 (0.0652–0.0735)	337	26	0.96 (0.56–1.63)	1.05 (0.61–1.81)	0.97 (0.55–1.72)
Q2 (0.0569–0.0651)	336	42	1.42 (0.88–2.30)	1.57 (0.96–2.55)	1.48 (0.90–2.44)
Q1 (0.0068–0.0568)	336	55	1.98 (1.25–3.13)	2.23 (1.39–3.58)	2.07 (1.27–3.35)
P for trend			<0.001	<0.001	<0.001
**P‐τ181 (pg/mL)**					
Q1 (0.381–3.198)	336	29	1.00 (reference)	1.00 (reference)	1.00 (reference)
Q2 (3.199–4.124)	337	25	0.82 (0.48–1.41)	0.82 (0.48–1.40)	0.82 (0.47–1.42)
Q3 (4.125–5.465)	337	41	1.07 (0.66–1.74)	1.09 (0.67–1.77)	0.996 (0.60–1.65)
Q4 (5.466–44.946)	336	56	1.35 (0.85–2.14)	1.37 (0.86–2.18)	1.33 (0.83–2.13)
P for trend			0.09	0.08	0.12
**GFAP (pg/mL)**					
Q1 (29.723–126.538)	336	12	1.00 (reference)	1.00 (reference)	1.00 (reference)
Q2 (126.539–165.624)	337	24	1.71 (0.85–3.43)	1.86 (0.92–3.74)	1.87 (0.90–3.86)
Q3 (165.625–227.046)	337	39	2.24 (1.15–4.36)	2.41 (1.23–4.71)	2.53 (1.26–5.07)
Q4 (227.047–1095.508)	336	76	3.72 (1.94–7.14)	3.98 (2.06–7.71)	4.27 (2.14–8.48)
*P* for trend			<0.001	<0.001	<0.001
**NfL (pg/mL)**					
Q1 (7.131–21.681)	336	9	1.00 (reference)	1.00 (reference)	1.00 (reference)
Q2 (21.682–28.780)	337	26	2.50 (1.17–5.37)	2.56 (1.19–5.49)	2.80 (1.25–6.25)
Q3 (28.781–39.271)	337	42	3.64 (1.74–7.60)	3.78 (1.81–7.92)	4.18 (1.92–9.09)
Q4 (39.272–675.691)	336	74	4.18 (1.97–8.88)	4.05 (1.90–8.63)	4.61 (2.08–10.22)
*P* for trend			<0.001	<0.001	<0.001

Aβ42/40, amyloid β42/40 ratio; CI, confidence interval; GFAP, glial fibrillary acid protein; NfL, neurofilament light chain; p‐τ181, phosphorylated tau (p‐τ)181; Q1, quartile 1; Q2, quartile 2; Q3, quartile 3; Q4, quartile 4.

^†^ Adjusted for age, sex, low education, hypertension, diabetes, body mass index, history of stroke, smoking habits, and sedentariness.

^‡^ Adjusted for age, sex, low education, hypertension, diabetes, estimated glomerular filtration rate, body mass index, history of stroke, smoking habits, sedentariness, and APOE‐ε4.

Regarding the association of each plasma biomarker level with the risk of dementia subtypes (Table 4), the risks of AD, but not non‐AD, increased significantly with lower plasma Aβ42/40 and higher plasma p‐τ181 levels even after multivariable adjustment (all *P* for trend <0.05). On the other hand, there was a significant association between higher plasma GFAP levels and risk of developing both AD and non‐AD (all *P* for trend <0.05). A similar significant association was observed between higher plasma NfL levels and risk of both AD and non‐AD (all *P* for trend <0.05). In the sensitivity analyses, the observed associations of each plasma biomarker with the risk of all‐cause dementia, AD, and non‐AD did not change substantially after dividing participants into quintiles and excluding participants with MCI and those with an MMSE score of <24 at baseline, respectively (Tables S2–S4). Moreover, Table S5 shows the association between each plasma biomarker level and a decrease in the MMSE score of five or more points from 2012 to 2017, after excluding 249 participants who were unable to undergo the MMSE in 2017 because they died or moved to a different town during the follow‐up. The multivariable‐adjusted risk for a decrease of five or more points in the MMSE score increased significantly with lower plasma Aβ42/40 levels and higher GFAP and NfL levels, but no such association was observed for plasma p‐τ181 levels.

**Table 4:** Association of plasma quartile levels of each biomarker with the risk of developing AD and non‐AD: 2012–2017

		AD				Non‐AD			
			Hazard ratio (95% CI)				Hazard ratio (95% CI)		
Plasma biomarker levels	Persons at risk	No. of events	Age‐ and sex‐adjusted	Multivariable‐adjusted^†^	Multivariable‐adjusted^‡^	No. of events	Age‐ and sex‐adjusted	Multivariable‐adjusted^†^	Multivariable‐adjusted^‡^
**Aβ42/40**									
Q4 (0.0736–0.2172)	336	20	1.00 (reference)	1.00 (reference)	1.00 (reference)	8	1.00 (reference)	1.00 (reference)	1.00 (reference)
Q3 (0.0652–0.0735)	337	15	0.75 (0.38–1.47)	0.81 (0.41–1.60)	0.77 (0.38–1.57)	11	1.52 (0.61–3.79)	1.67 (0.65–4.31)	1.42 (0.54–3.75)
Q2 (0.0569–0.0651)	336	30	1.42 (0.81–2.50)	1.55 (0.87–2.75)	1.52 (0.84–2.74)	12	1.43 (0.58–3.49)	1.53 (0.60–3.89)	1.40 (0.55–3.59)
Q1 (0.0068–0.0568)	336	43	2.20 (1.29–3.76)	2.38 (1.38–4.10)	2.24 (1.27–3.94)	12	1.45 (0.59–3.56)	1.79 (0.69–4.62)	1.62 (0.63–4.17)
*P* for trend			<0.001	<0.001	<0.001		0.49	0.28	0.35
**P‐**τ**181 (pg/mL)**									
Q1 (0.381–3.198)	336	17	1.00 (reference)	1.00 (reference)	1.00 (reference)	8	1.00 (reference)	1.00 (reference)	1.00 (reference)
Q2 (3.199–4.124)	337	19	1.07 (0.55–2.05)	1.07 (0.55–2.05)	0.99 (0.50–1.94)	11	1.52 (0.61–3.79)	1.67 (0.65–4.31)	0.53 (0.20–1.45)
Q3 (4.125–5.465)	337	31	1.40 (0.77–2.55)	1.43 (0.78–2.62)	1.23 (0.66–2.28)	12	1.43 (0.58–3.49)	1.53 (0.60–3.89)	0.62 (0.25–1.51)
Q4 (5.466–44.946)	336	41	1.73 (0.97–3.09)	1.78 (0.994–3.17)	1.69 (0.94–3.03)	12	1.45 (0.59–3.56)	1.79 (0.69–4.62)	0.74 (0.32–1.71)
*P* for trend			0.03	0.03	0.04		0.49	0.28	0.60
**GFAP (pg/mL)**									
Q1 (29.723–126.538)	336	9	1.00 (reference)	1.00 (reference)	1.00 (reference)	3	1.00 (reference)	1.00 (reference)	1.00 (reference)
Q2 (126.539–165.624)	337	16	1.51 (0.67–3.43)	1.59 (0.70–3.63)	1.61 (0.68–3.84)	8	2.33 (0.61–8.82)	2.71 (0.71–10.35)	2.68 (0.70–10.28)
Q3 (165.625–227.046)	337	30	2.24 (1.04–4.82)	2.44 (1.12–5.28)	2.63 (1.17–5.89)	9	2.23 (0.58–8.56)	2.04 (0.52–7.97)	2.18 (0.55–8.67)
Q4 (227.047–1095.508)	336	53	3.24 (1.52–6.92)	3.57 (1.66–7.68)	3.74 (1.67–8.38)	23	5.35 (1.48–19.40)	5.01 (1.36–18.48)	5.54 (1.50–20.52)
*P* for trend			<0.001	<0.001	<0.01		0.004	0.01	<0.01
**NfL (pg/mL)**									
Q1 (7.131–21.681)	336	6	1.00 (reference)	1.00 (reference)	1.00 (reference)	3	1.00 (reference)	1.00 (reference)	1.00 (reference)
Q2 (21.682–28.780)	337	19	2.67 (1.06–6.72)	2.69 (1.07–6.78)	3.18 (1.18–8.60)	7	2.18 (0.56–8.52)	2.24 (0.57–8.77)	2.14 (0.53–8.71)
Q3 (28.781–39.271)	337	34	4.20 (1.73–10.21)	4.44 (1.82–10.80)	5.07 (1.94–13.27)	8	2.37 (0.61–9.24)	2.30 (0.58–9.06)	2.52 (0.64–9.98)
Q4 (39.272–675.691)	336	49	3.83 (1.53–9.60)	4.00 (1.59–10.10)	4.56 (1.68–12.38)	25	5.19 (1.39–19.37)	3.83 (1.02–14.39)	4.83 (1.28–18.24)
*P* for trend			0.004	0.003	0.003		0.007	0.04	0.01

Aβ42/40, amyloid β42/40 ratio; AD, Alzheimer disease; CI, confidence interval; eGFR, estimated glomerular filtration rate; non‐AD, non–Alzheimer dementia; P‐τ, phosphorylated tau.

^†^ Adjusted for age, sex, low education, hypertension, diabetes, body mass index, history of stroke, smoking habits, and sedentariness.

^‡^ Adjusted for age, sex, low education, hypertension, diabetes, estimated glomerular filtration rate, body mass index, history of stroke, smoking habits, sedentariness, and APOE‐ε4.

**Fig. 1:**
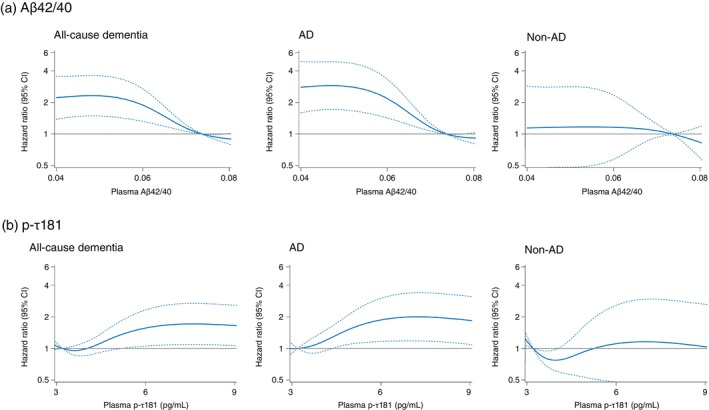
Restricted cubic spline for the association of plasma levels of amyloid β42/40 ratio (Aβ42/40; A) and phosphorylated tau (p‐τ)181; B) with the risk of all‐cause dementia and its subtypes. Solid lines represent the hazard ratios; dashed lines represent the 95% confidence intervals (CIs). Knots were placed at the fifth, 35th, 65th, and 95th percentiles of plasma Aβ42/40 and p‐τ181 (0.03995, 0.06060, 0.07016, and 0.08596 for Aβ42/40; and 2.298, 3.572, 4.845, and 9.099 pg/mL for p‐τ181, respectively). The fifth percentile of each plasma biomarker was set as the reference value. The risk estimates were adjusted for age, sex, low education, hypertension, diabetes, estimated glomerular filtration rate, body mass index, history of stroke, smoking habits, sedentariness, and APOE‐ε4.

**Fig. 2:**
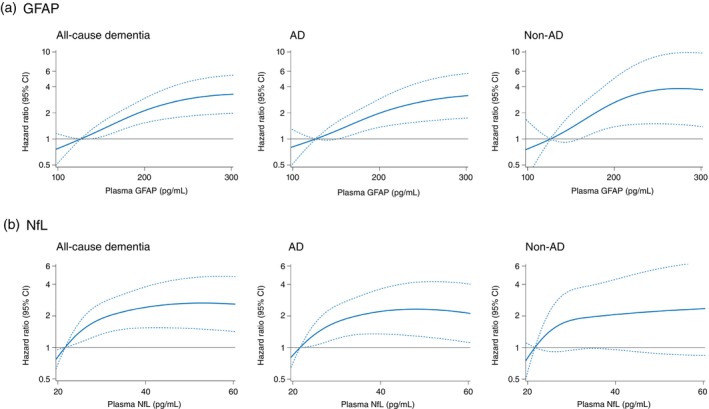
Restricted cubic spline for the association of plasma levels of glial fibrillary acidic protein (GFAP; A) and neurofilament light chain (NfL; B) with the risk of all‐cause dementia and its subtypes. Solid lines represent the hazard ratios; dashed lines represent the 95% confidence intervals (CIs). Knots were placed at the fifth, 35th, 65th, and 95th percentiles of plasma GFAP and NfL (85.531, 140.835, 197.308, and 376.407 pg/mL for GFAP; and 15.114, 24.159, 34.191, and 77.594 pg/mL for NfL, respectively). The fifth percentile of each plasma biomarker was set as the reference value. The risk estimates were adjusted for age, sex, low education, hypertension, diabetes, estimated glomerular filtration rate, body mass index, history of stroke, smoking habits, sedentariness, and APOE‐ε4.

To assess the shape of the association between plasma Aβ42/40, p‐τ181, GFAP, and NfL levels and risk of all‐cause dementia, we used a restricted cubic spline analysis (Figs 1 and 2). The risk of all‐cause dementia increased approximately linearly with lower plasma Aβ42/40 levels between around 0.075 to 0.060 and then plateaued thereafter (*P* for nonlinearity = 0.02) (Fig. 1a). The risk of all‐cause dementia was relatively flat at the low end of the plasma p‐τ181 levels, but increased rapidly between around 4 to 8 pg/mL and then plateaued thereafter (*P* for nonlinearity = 0.02) (Fig. 1b). Higher plasma GFAP levels showed an almost linear positive association with the risk of all‐cause dementia around a plasma GFAP level of 300 pg/mL and then plateaued (*P* for nonlinearity = 0.03) (Fig. 2a). Similarly, the risk of all‐cause dementia increased approximately linearly with higher levels of NfL around 35 pg/mL and plateaued thereafter (*P* for nonlinearity <0.01) (Fig. 2b). With regard to the subtypes of dementia, the associations between each plasma biomarker and risk of developing AD were similar to those seen for the risk of developing total dementia (Figs 1 and 2). On the other hand, the risk of developing non‐AD increased with higher plasma levels of GFAP and NfL, similar to the association with all‐cause dementia, but no clear association was found between plasma levels of Aβ42/40 or p‐τ181 and the risk of non‐AD (Figs 1 and 2).

Finally, we assessed the discrimination and reclassification ability of each plasma biomarker for the development of all‐cause dementia. The C statistic, cutoff value, and HR of each plasma biomarker for incident all‐cause dementia are shown in Table S6. The C statistics and cutoff value of each plasma biomarker were as follows: 0.592 (0.542–0.641), 0.0591 for plasma Aβ42/40; 0.593 (0.544–0.642), 4.625 pg/mL for plasma p‐τ181; 0.709 (0.666–0.752), 186.60 pg/mL for plasma GFAP; and 0.699 (0.659–0.741), 27.10 pg/mL for plasma NfL. In addition, we investigated the predictive ability of the plasma biomarkers for incident dementia by adding each plasma biomarker to the predicted dementia risk calculated by the risk score consisting of known risk factors for dementia in the clinical prediction model (Table 5). As compared with the C statistics of the predicted dementia risk alone (C statistic: 0.727), the C statistics of the model with addition of plasma Aβ42/40 achieved marginally significant improvement in discrimination (C statistic: 0.742; *P for difference in C statistics* = 0.05). When plasma p‐τ181 was incorporated into the predicted dementia risk, no significant increment of C statistics was observed (C statistic: 0.731; *P for difference in C statistics* = 0.21). On the other hand, the model adding plasma GFAP or NfL levels to the predicted dementia risk showed a significant improvement in discrimination, respectively (for GFAP: C statistic = 0.757; *P for difference in C statistics* < 0.01; for NfL: C statistic = 0.736; *P for difference in C statistics* < 0.01). We further confirmed that the discrimination of the predicted dementia risk was significantly improved by adding plasma Aβ42/40 and GFAP simultaneously to the predicted dementia risk (C statistic: 0.764; *P for difference in C statistics* < 0.01). Similar significant improvements of predictive ability were observed for the model consisting of the predicted dementia risk plus Aβ42/40 + GFAP + NfL and the model consisting of the predicted dementia risk plus Aβ42/40 + GFAP + NfL + p‐τ181, respectively. Moreover, we further confirmed the significant improvement of continuous NRI and IDI by adding each plasma biomarker, except for plasma NfL alone, to the model consisting of the predicted dementia risk. A sensitivity analysis assessing the changes in the risk assessment ability for incident dementia by adding each plasma biomarker divided into binary variables at the cutoff values to the predicted dementia risk calculated by the risk score consisting of known risk factors for dementia showed that these additions made little or no change to the significant improvement of predictive ability for incident dementia shown in Table 5 (Table S7).

**Table 5:** Changes in the risk assessment ability for developing dementia by adding each plasma biomarker to the predicted dementia risk calculated by the risk score consisting of known risk factors for dementia: 2012–2017

	C statistic for developing dementia (95% CI)	*P* value for difference in C statistics	Continuous NRI for developing dementia (95% CI)	*P* value for NRI	IDI for developing dementia (95% CI)	*P* value for IDI
Predicted dementia risk	0.727 (0.689–0.765)					
Predicted dementia risk + Aβ42/40	0.742 (0.705–0.780)	0.05	0.336 (0.170–0.503)	<0.001	0.008 (0.0001–0.014)	0.02
Predicted dementia risk + p‐τ181	0.731 (0.693–0.769)	0.21	0.213 (0.047–0.380)	0.01	0.0002 (−0.0009–0.001)	0.73
Predicted dementia risk + GFAP	0.757 (0.721–0.793)	0.002	0.328 (0.160–0.496)	<0.001	0.016 (0.0048–0.0276)	0.005
Predicted dementia risk + NfL	0.736 (0.699–0.774)	0.002	−0.066 (−0.228–0.096)	0.44	−0.00002 (−0.002–0.002)	0.98
Predicted dementia risk + Aβ42/40 + GFAP	0.764 (0.728–0.799)	0.002	0.411 (0.243–0.578)	<0.001	0.021 (0.008–0.034)	0.001
Predicted dementia risk + Aβ42/40 + GFAP + NfL	0.763 (0.727–0.799)	0.002	0.421 (0.253–0.588)	<0.001	0.022 (0.009–0.035)	0.001
Predicted dementia risk + Aβ42/40 + GFAP + NfL + p‐τ18	0.765 (0.729–0.800)	0.001	0.447 (0.280–0.614)	<0.001	0.022 (0.009–0.035)	<0.001

The predicted dementia risk was calculated based on the previously developed clinical prediction model for the development of dementia consisting of the following variables: age, female, low education, hypertension, diabetes, body mass index, history of stroke, current smoking, and sedentariness (Honda et al.^35^).

Aβ42/40, amyloid β42/40 ratio; CI, confidence interval; GFAP, glial fibrillary acid protein; IDI, integrated discrimination improvement; NfL, neurofilament light chain; NRI, net reclassification improvement; p‐τ181, phosphorylated tau (p‐τ)181.

## Discussion

In this prospective cohort study of Japanese older residents without dementia, lower plasma Aβ42/40 levels and higher plasma p‐τ181 levels were significantly associated with an increased risk of AD but not non‐AD, whereas significant associations were observed between higher plasma GFAP and NfL levels and risk of developing both AD and non‐AD. These associations did not change when excluding participants with MCI and those with an MMSE score of <24 at baseline. In addition, the ability of the model to predict the risk of developing dementia was improved by adding these plasma biomarkers. These findings suggest that plasma Aβ42/40, p‐τ181, GFAP, and NfL may be effective plasma biomarkers for identifying participants at high risk for the development of dementia in clinical settings.

Plasma Aβ42/40 has been considered to be a reliable biomarker of the neocortical Aβ burden. Several clinical and population‐based studies found a significant association between lower plasma Aβ42/40 levels and risk of dementia, especially AD.^5^, ^6^, ^7^, ^8^, ^9^, ^10^ In addition, several prospective studies assessed the association between plasma p‐τ181 levels and risk of dementia, and all these studies found that an increased plasma p‐τ181 level was a significant specific risk factor for incident AD.^9^, ^11^, ^12^ Meanwhile, no prospective studies have assessed the association between plasma GFAP levels and risk of dementia. In regard to serum samples, the Chicago Health and Aging Project, a population‐based prospective study of Canadian residents,^15^ and the Amsterdam Dementia Cohort, a clinic‐based prospective study,^16^ reported a significant association between higher serum GFAP levels and the risk of developing AD and all‐cause dementia, respectively. For plasma NfL, the Rotterdam Study, the Chicago Health and Aging Project, and the Amsterdam Dementia Cohort showed a significant association between higher plasma or serum NfL levels and an increased risk of all‐cause dementia and AD, respectively.^8^, ^15^, ^16^ These previous findings support the results of the present study, suggesting that there is a significant association between plasma Aβ42/40, p‐τ181, GFAP, or NfL levels and the risk of developing dementia in the general older population.

The present findings showed the nonlinear nature of the association between the plasma p‐τ181, GFAP, and NfL concentrations and risk of all‐cause dementia, with an exposure–response association being observed within a range of relatively low to intermediate levels of each plasma biomarker, and the risk of dementia reaching a plateau at the high end of each plasma biomarker level and at the low end for Aβ42/40. When considering the A‐T‐N neuropathological cascade of developing dementia,^2^ these results may imply that such low plasma Aβ42/40 levels or high p‐τ181 and NfL levels cause neurodegeneration or neuroaxonal brain injury and were sufficiently increased to exert a maximum influence on dementia risk, especially in the case of NfL.^4^ The same may also be true for the association with GFAP, because the concentrations of plasma GFAP have been reported to increase linearly with higher levels of Aβ burden, preceded by an increase in plasma p‐τ181, and then to plateau with a high Aβ burden.^4^, ^14^ However, for the association of the plasma Aβ42/40–GFAP–p‐τ–NfL cascade with the development of dementia, we cannot rule out the possibility that our observation of plateaus in the associations between these plasma biomarkers and the risk of dementia does not accurately reflect the real‐life relations due to the relatively short term of follow‐up. To elucidate the role of the Aβ42/40–GFAP–p‐τ–NfL cascade in the development of dementia, we plan to conduct a future population‐based prospective study with a larger scale and longer follow‐up periods than this study.

In this study, plasma Aβ42/40 and p‐τ181 levels were significantly associated with the risk of developing AD but not non‐AD. As mentioned above, clinical and prospective studies have also shown the significant association between lower plasma Aβ42/40 levels and higher risk of AD.^5^, ^6^, ^7^, ^9^, ^10^ The concentrations of plasma p‐τ181 have been reported to be strongly increased in individuals with clinically diagnosed AD,^40^ and, in other studies, these associations were observed only in individuals with Aβ pathological changes,^4^, ^41^ which supports the idea that plasma p‐τ181 is a specific marker for the neuropathological changes of AD. On the other hand, the present study showed significant associations of higher plasma GFAP and NfL levels with risk of developing not only AD but also non‐AD, and plasma GFAP and NfL had higher C statistics for detecting incident dementia than plasma Aβ42/40 levels and plasma p‐τ181. Moreover, a significant improvement of the risk assessment ability for incident dementia was observed when adding plasma GFAP or NfL alone to the predicted dementia risk calculated by the risk score consisting of known risk factors for dementia. Clinical studies showed a sharp and sustained increase in plasma GFAP as soon as CSF Aβ and amyloid PET became positive,^4^ followed by a slightly delayed, smaller increase in plasma p‐τ181,^14^ possibly suggesting that GFAP reflects the brain changes related to Aβ deposition. On the other hand, GFAP is known as a cytoskeletal component of astrocytes,^13^ and astrocyte activation has been implicated as a potential driver or effect of pathological changes of both AD and non‐AD, because clinical studies showed that patients with non‐AD, such as dementia with Lewy bodies and frontotemporal degeneration, also had higher CSF, plasma, and serum GFAP levels than those with normal cognition.^4^, ^42^ These findings suggest that GFAP may be a potential biomarker for the brain changes related to not only AD but also non‐AD. Similarly, NfL is a component of the axonal cytoskeleton and known as a marker of neurodegeneration, neuroaxonal injury, or vascular diseases, irrespective of the underlying cause.^4^, ^8^, ^15^, ^42^ Our findings of an association of plasma NfL with the risk of both AD and non‐AD are in accordance with these previous data. In addition, the present study showed that higher levels of plasma NfL were significantly associated with higher frequencies of history of stroke, history of cerebrocardiovascular disease, and sedentariness. Our results indicate that higher plasma NfL levels may represent the accumulation of neurodegeneration and vascular damage in the brain and therefore may be linked to higher frequency of sedentariness. Taken together, these findings raise the possibility that plasma Aβ42/40 and p‐τ181 may be specific markers for the development of AD, while plasma GFAP and NfL may be potential biomarkers for the development of not only AD but also other types of dementia as well. Nevertheless, the discrepancies among these plasma biomarkers in the association with dementia may derive from the limited number of events for each subtype of dementia, or may reflect the differences in the timing of changes for each biomarker in the course of dementia onset. Further large‐scale and long‐term prospective studies are warranted to assess the dementia risk associated with high plasma levels of each biomarker in greater detail.

The strengths of our study are the population‐based prospective cohort study design, the consistent and detailed methods of detection and diagnosis of dementia cases in follow‐up surveys, the thorough follow‐up of participants, and the detailed evaluation of known risk factors. However, several limitations also bear mention. First, there was a possibility of selection bias caused by excluding individuals without stored plasma samples. However, the participants excluded from this study were significantly older and had significantly lower scores of the MMSE and the Barthel index than those included in this study (data not shown), which could have weakened the association between plasma biomarkers and dementia risk. Second, individuals in the prodromal stage of dementia might have been included at baseline. However, our sensitivity analyses excluding participants with MCI and those with an MMSE score of <24 at baseline did not alter the findings of this study. Third, since the participants of this study were from one town in Japan, the generalizability of our findings to populations with different ethnicities may be limited. Fourth, we cannot rule out possible residual confounding by unmeasured confounders (e.g. traumatic injury). Fifth, we could not quantify plasma levels of p‐τ217, which was reported to be more strongly associated with Alzheimer pathology than p‐τ181,^4^ because an assay kit for plasma p‐τ217 was not available.

In conclusion, our data suggest that plasma Aβ42/40 and p‐τ181 are specific plasma biomarkers for the development of AD, and plasma GFAP and NfL are potential biomarkers for all‐cause dementia in the general older population. In addition, the predictive ability for development of dementia is significantly improved when adding plasma Aβ42/40, p‐τ181, GFAP, and NfL levels into a model consisting of established risk factors. These findings suggest that the measurement of these plasma biomarkers may be a useful and relatively less invasive procedure for identifying individuals at high risk for developing dementia in a clinical setting. Further large‐scale and long‐term follow‐up studies are required to verify the findings of the present study.

## Funding

This study was supported in part by the Ministry of Education, Culture, Sports, Science and Technology of Japan (JSPS KAKENHI grant numbers JP21H03200, JP21K07522, JP21K11725, JP21K10448, JP22K07421, JP22K17396, JP23K09692, JP23K09717, JP23K16330, JP23K06787, and JP23K09060); by the Health and Labor Sciences Research Grants of the Ministry of Health, Labor and Welfare of Japan (grant numbers JPMH23FA1006 and JPMH23FA1022); by the Japan Agency for Medical Research and Development (numbers JP21dk0207055, JP22dk0207053, and JP23km0405209); and by a JST Grant (number JPMJPF2210).

## Disclosure statement

TO and MM are editorial board members of *Psychiatry and Clinical Neurosciences* and coauthors of this article. To minimize bias, they were excluded from all editorial decision‐making related to the acceptance of this article for publication. T. Maeda reports honoraria for lectures from Eisai, Sumitomo Pharma, Ono Pharma, Biogen Japan, Kowa, Daiichi Sankyo, and Novartis Pharma. HT, JH, TH, MS, SM, T. Mikami, KO, KN, JI, MT, TT, and TN report no disclosures.

## Author contributions

TO and TN contributed to the study conception and study design; TO, JH, TH and TN contributed to the data analysis; TO, HT, JH, TH, MS, SM, TT, and TN contributed to the data collection; TO, HT, JH, TH, MS, SM, T Mikami, T Maeda, KO, MM, KN, JI, MT, TT, and TN contributed to the data interpretation. TO and TN wrote the first draft of the manuscript and all authors contributed to critical revision of the manuscript.

## Supporting information


**Fig. S1:** Correlation plots between each log‐transformed plasma biomarker (plasma levels of amyloid β42/40 [A]; phosphorylated tau (p‐τ)181 [B]) and age, sex, and the Mini‐Mental State Examination (MMSE).
**Fig. S2:** Correlation plots between each log‐transformed plasma biomarker (plasma levels of glial fibrillary acidic protein [A] and neurofilament light chain [B]) and age, sex, and the Mini‐Mental State Examination (MMSE).

**Table S1.** Correlation coefficient between plasma biomarkers: 2012–2013.
**Table S2.** Multivariable‐adjusted risk of all‐cause dementia and its subtypes according to plasma quintile levels of each dementia biomarker: 2012–2017.
**Table S3.** Multivariable‐adjusted risk of all‐cause dementia and its subtypes according to plasma quartile levels of each dementia biomarker after excluding participants with mild cognitive impairment (MCI): 2012–2017.
**Table S4.** Multivariable‐adjusted risk of all‐cause dementia and its subtypes according to plasma quartile levels of each dementia biomarker after excluding participants with Mini‐Mental State Examination (MMSE) <24: 2012–2017.
**Table S5.** Multivariable‐adjusted odds ratio for a decrease of five or more points in the Mini‐Mental State Examination (MMSE) score from 2012 to 2017 according to plasma quartile levels of each biomarker: 2012–2017.
**Table S6.** C statistics of each plasma biomarker for incident dementia and the multivariable‐adjusted risk for incident dementia in participants with a plasma level below the cutoff value for the plasma amyloid β42/40 ratio or above the cutoff level for phosphorylated tau (p‐τ)181, glial fibrillary acid protein, or neurofilament light chain: 2012–2017.
**Table S7.** Changes in the risk assessment ability for incident dementia by adding each plasma biomarker divided into binary variables at the cutoff values shown in Table S6 to the predicted dementia risk calculated by the risk score consisting of known risk factors for dementia: 2012–2017.

**Data S1:** Supplementary Data.

**Data S2:** STROBE (Strengthening the Reporting of Observational Studies in Epidemiology) checklist cohort.

## Data Availability

The data sets used in the present study are not publicly available because they contain confidential clinical data on the study participants. However, the data are available on reasonable request and with the permission of the principal investigator of this study, Toshiharu Ninomiya.
