# Supplementary material for: Plasma biomarkers for predicting the development of dementia in a community‐dwelling older Japanese population
Source: Psychiatry Clin Neurosci. 2024 Apr 12;78(6):362–71. doi: 10.1111/pcn.13661 (PMC11488610; doi:10.1111/pcn.13661)
Supplement: Supplementary file 1 — Fig. S1: Correlation plots between each log‐transformed plasma biomarker (plasma levels of amyloid β42/40 [A]; phosphorylated tau (p‐τ)181 [B]) and age, sex, and the Mini‐Mental State Examination (MMSE). Fig. S2: Correlation plots between each log‐transformed plasma biomarker (plasma levels of glial fibrillary acidic protein [A] and neurofilament light chain [B]) and age, sex, and the Mini‐Mental State Examination (MMSE). [file PCN-78-362-s001.pptx]

## Slide 1
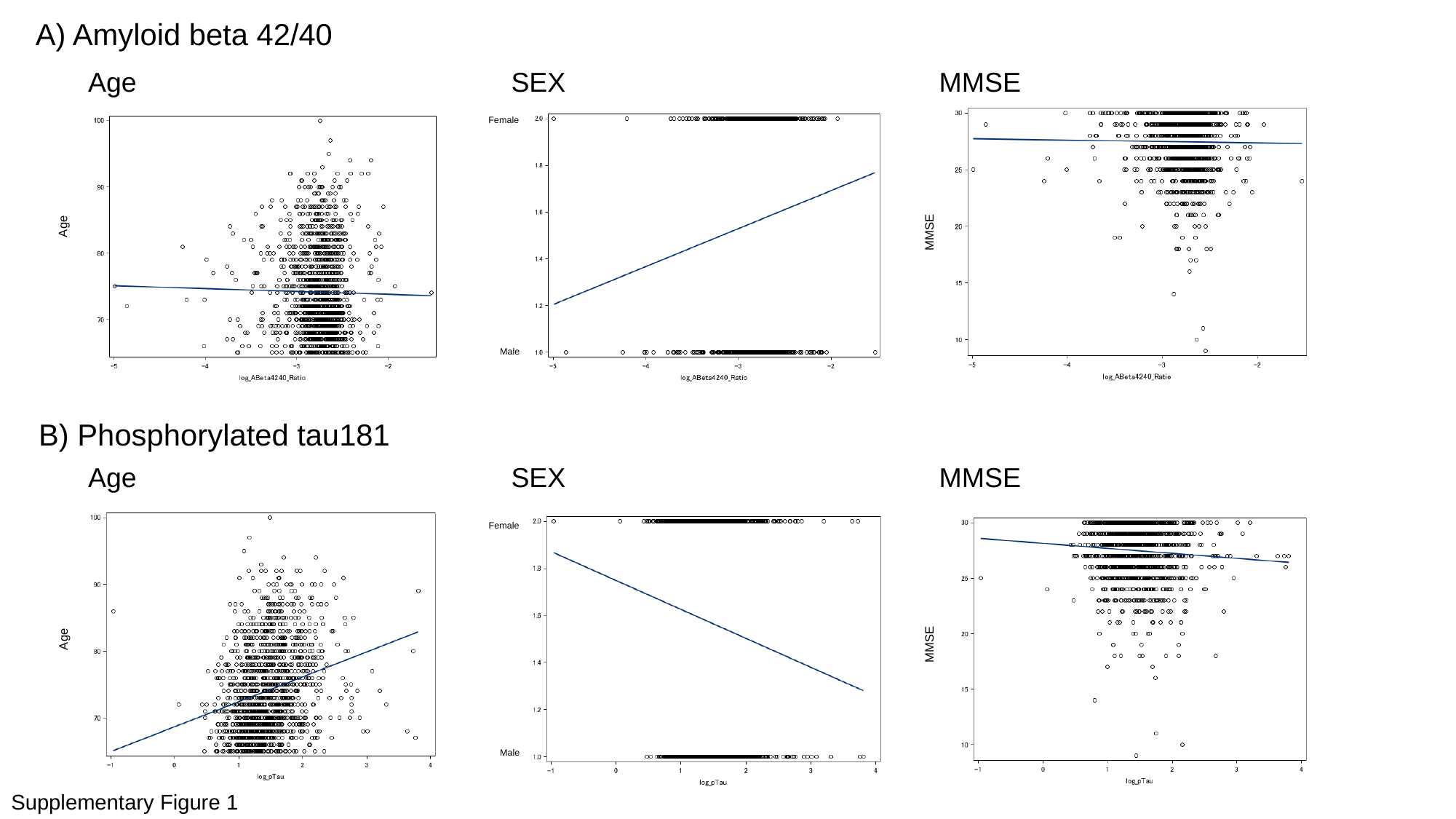

A) Amyloid beta 42/40
Age
SEX
MMSE
Female
MMSE
Age
Male
B) Phosphorylated tau181
SEX
MMSE
Age
Female
MMSE
Age
Male
Supplementary Figure 1

## Slide 2
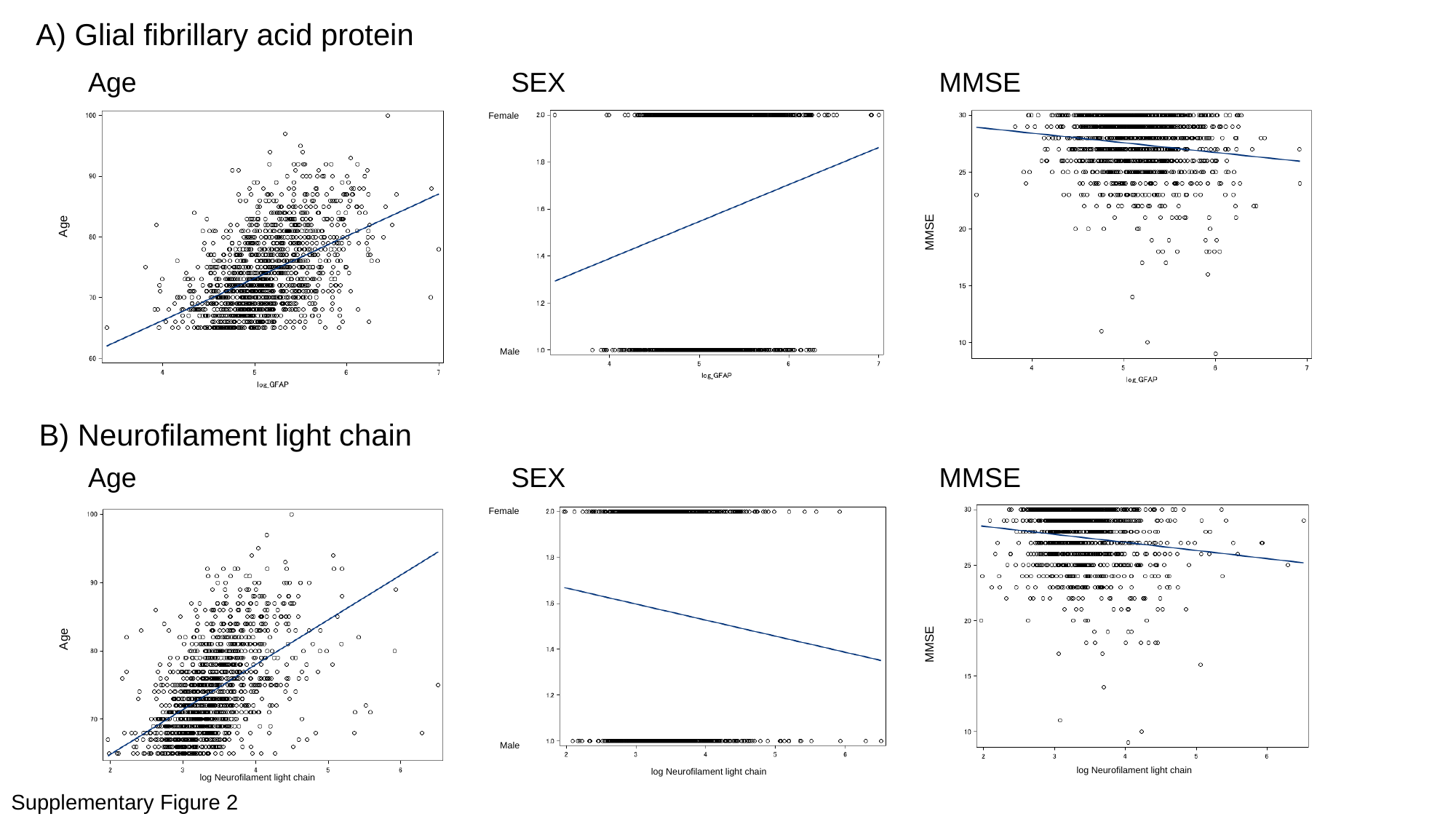

A) Glial fibrillary acid protein
Age
SEX
MMSE
Female
MMSE
Age
Male
B) Neurofilament light chain
SEX
MMSE
Age
Female
MMSE
Age
Male
log Neurofilament light chain
log Neurofilament light chain
log Neurofilament light chain
Supplementary Figure 2
