# Supplementary material for: Plasma biomarkers for predicting the development of dementia in a community‐dwelling older Japanese population
Source: Psychiatry Clin Neurosci. 2024 Apr 12;78(6):362–71. doi: 10.1111/pcn.13661 (PMC11488610; doi:10.1111/pcn.13661)
Supplement: Supplementary file 2 — Table S1. Correlation coefficient between plasma biomarkers: 2012–2013. Table S2. Multivariable‐adjusted risk of all‐cause dementia and its subtypes according to plasma quintile levels of each dementia biomarker: 2012–2017. Table S3. Multivariable‐adjusted risk of all‐cause dementia and its subtypes according to plasma quartile levels of each dementia biomarker after excluding participants with mild cognitive impairment (MCI): 2012–2017. Table S4. Multivariable‐adjusted risk of all‐cause dementia and its subtypes according to plasma quartile levels of each dementia biomarker after excluding participants with Mini‐Mental State Examination (MMSE) <24: 2012–2017. Table S5. Multivariable‐adjusted odds ratio for a decrease of five or more points in the Mini‐Mental State Examination (MMSE) score from 2012 to 2017 according to plasma quartile levels of each biomarker: 2012–2017. Table S6. C statistics of each plasma biomarker for incident dementia and the multivariable‐adjusted risk for incident dementia in participants with a plasma level below the cutoff value for the plasma amyloid β42/40 ratio or above the cutoff level for phosphorylated tau (p‐τ)181, glial fibrillary acid protein, or neurofilament light chain: 2012–2017. Table S7. Changes in the risk assessment ability for incident dementia by adding each plasma biomarker divided into binary variables at the cutoff values shown in Table S6 to the predicted dementia risk calculated by the risk score consisting of known risk factors for dementia: 2012–2017. [file PCN-78-362-s003.docx]

| **Supplementary table 1. Correlation coefficient between plasma biomarkers, 2012-2013** | | | | |
| --- | --- | --- | --- | --- |
|  | **Aβ42/40** | **Phosphorylated tau181** | **Glial fibrillary acid protein** | **Neurofilament light chain** |
| **Aβ42/40** | 1.00  (reference) | -0.18  p = <0.0001 | -0.13  p = <0.0001 | -0.06  p = 0.03 |
| **Phosphorylated tau181 (pg/mL)** | -0.18  p = <0.0001 | 1.00  (reference) | 0.33  p = <0.001 | 0.38  p = <0.001 |
| **Glial fibrillary acid protein (pg/mL)** | -0.13  p = <0.0001 | 0.33  p = <0.001 | 1.00  (reference) | 0.59  p = <0.001 |
| **Neurofilament light chain (pg/mL)** | -0.06  p = 0.03 | 0.38  p = <0.001 | 0.59  p = <0.001 | 1.00  (reference) |
| Aβ42/40, amyloid beta 42/40 ratio | | | | |

| **Supplementary table 2. Multivariable-adjusted risk of all-cause dementia and its subtypes according to plasma quintile levels of each biomarker, 2012–2017** | | | | |
| --- | --- | --- | --- | --- |
| **Plasma biomarker levels** | **Hazard ratio (95% CI) ^a)^** | | |  |
|  | **All-cause dementia** | **Alzheimer’s disease** | **Non-Alzheimer’s dementia** |  |
| **Aβ42/40** |  |  |  |  |
| Q5 (0.0754-0.2172) | 1.00 (reference) | 1.00 (reference) | 1.00 (reference) |  |
| Q4 (0.0686-0.0754) | 1.07 (0.57-1.98) | 1.13 (0.54-2.37) | 0.88 (0.28-2.76) |  |
| Q3 (0.0620-0.0686) | 1.06 (0.58-1.94) | 0.90 (0.41-1.95) | 1.24 (0.45-3.39) |  |
| Q2 (0.0546-0.0620) | 1.92 (1.11-3.32) | 2.13 (1.11-4.08) | 1.49 (0.53-4.20) |  |
| Q1 (0.0068-0.0546) | 2.07 (1.20-3.54) | 2.43 (1.29-4.58) | 1.25 (0.43-3.63) |  |
| P for trend | <0.001 | <0.001 | 0.42 |  |
|  |  |  |  |  |
| **Phosphorylated tau181 (pg/mL)** |  |  |  |  |
| Q1 (0.381-3.023) | 1.00 (reference) | 1.00 (reference) | 1.00 (reference) |  |
| Q2 (3.024-3.775) | 0.68 (0.37-1.26) | 0.83 (0.38-1.81) | 0.45 (0.16-1.25) |  |
| Q3 (3.776-4.532) | 0.93 (0.52-1.65) | 1.34 (0.66-2.72) | 0.38 (0.12-1.16) |  |
| Q4 (4.533-5.896) | 0.93 (0.53-1.62) | 1.06 (0.52-2.18) | 0.68 (0.28-1.68) |  |
| Q5 (5.897-44.946) | 1.19 (0.69-2.05) | 1.81 (0.93-3.55) | 0.38 (0.14-1.06) |  |
| P for trend | 0.21 | 0.04 | 0.25 |  |
|  |  |  |  |  |
| **Glial fibrillary acid protein (pg/mL)** |  |  |  |  |
| Q1 (29.72-117.86) | 1.00 (reference) | 1.00 (reference) | 1.00 (reference) |  |
| Q2 (117.87-147.75) | 2.08 (0.85-5.11) | 2.08 (0.72-5.96) | 2.15 (0.39-12.01) |  |
| Q3 (147.77-183.11) | 2.82 (1.18-6.74) | 2.46 (0.87-6.94) | 3.95 (0.79-19.69) |  |
| Q4 (183.19-245.72) | 4.15 (1.79-9.62) | 3.76 (1.39-10.16) | 5.09 (1.06-24.51) |  |
| Q5 (246.16-1095.51) | 6.61 (2.82-15.45) | 6.02 (2.22-16.35) | 7.98 (1.59-40.16) |  |
| P for trend | <0.001 | <0.001 | 0.003 |  |
|  |  |  |  |  |
| **Neurofilament light chain (pg/mL)** |  |  |  |  |
| Q1 (7.13-20.38) | 1.00 (reference) | 1.00 (reference) | 1.00 (reference) |  |
| Q2 (20.39-25.63) | 1.76 (0.71-4.38) | 2.21 (0.70-7.00) | 1.04 (0.21-5.20) |  |
| Q3 (25.64-32.13) | 3.36 (1.45-7.80) | 4.09 (1.39-12.08) | 2.23 (0.54-9.11) |  |
| Q4 (32.21-42.76) | 4.49 (1.94-10.40) | 5.50 (1.86-16.20) | 2.95 (0.74-11.82) |  |
| Q5 (42.84-675.69) | 4.37 (1.80-10.61) | 4.30 (1.36-13.63) | 4.55 (1.13-18.39) |  |
| P for trend | <0.001 | <0.001 | 0.009 |  |
| Abbreviations: CI, confidence interval; Aβ42/40, amyloid beta 42/40 ratio; eGFR, estimated glomerular filtration rate.   1. Adjusted for age, sex, low education, hypertension, diabetes, eGFR, body mass index, history of stroke, smoking habits, sedentariness, and APOE-ε4. | | | | |

| **Supplementary table 3. Multivariable-adjusted risk of all-cause dementia and its subtypes according to plasma quartile levels of each biomarker after excluding participants with MCI, 2012–2017** | | | | |
| --- | --- | --- | --- | --- |
| **Plasma biomarker levels** | **Hazard ratio (95% CI) ^a)^** | | |  |
|  | **All-cause dementia** | **Alzheimer’s disease** | **Non-Alzheimer’s dementia** |  |
| **Aβ42/40** |  |  |  |  |
| Q4 (0.0736-0.2172) | 1.00 (reference) | 1.00 (reference) | 1.00 (reference) |  |
| Q3 (0.0652-0.0735) | 0.93 (0.46-1.86) | 0.87 (0.34-2.23) | 0.93 (0.33-2.65) |  |
| Q2 (0.0595-0.0651) | 1.47 (0.79-2.72) | 1.63 (0.73-3.63) | 1.27 (0.48-3.40) |  |
| Q1 (0.0068-0.0594) | 2.48 (1.39-4.43) | 3.41 (1.63-7.12) | 1.25 (0.45-3.51) |  |
| P for trend | <0.001 | <0.001 | 0.56 |  |
|  |  |  |  |  |
| **Phosphorylated tau181 (pg/mL)** |  |  |  |  |
| Q1 (0.381-3.198) | 1.00 (reference) | 1.00 (reference) | 1.00 (reference) |  |
| Q2 (3.199-4.125) | 0.92 (0.47-1.80) | 1.21 (0.51-2.88) | 0.58 (0.19-1.74) |  |
| Q3 (4.126-5.465) | 1.13 (0.61-2.10) | 1.60 (0.73-3.52) | 0.60 (0.21-1.74) |  |
| Q4 (5.466-44.946) | 1.66 (0.92-2.99) | 2.31 (1.08-4.95) | 0.89 (0.33-2.38) |  |
| P for trend | 0.06 | 0.02 | 0.88 |  |
|  |  |  |  |  |
| **Glial fibrillary acid protein (pg/mL)** |  |  |  |  |
| Q1 (29.72-126.53) | 1.00 (reference) | 1.00 (reference) | 1.00 (reference) |  |
| Q2 (126.54-165.62) | 2.02 (0.81-5.06) | 1.34 (0.42-4.28) | 4.00 (0.82-19.62) |  |
| Q3 (165.63-227.04) | 3.06 (1.29-7.27) | 2.95 (1.06-8.22) | 3.21 (0.63-16.26) |  |
| Q4 (227.05-1095.51) | 6.12 (2.60-14.40) | 5.45 (1.97-15.07) | 7.19 (1.47-35.09) |  |
| P for trend | <0.001 | <0.001 | 0.01 |  |
|  |  |  |  |  |
| **Neurofilament light chain (pg/mL)** |  |  |  |  |
| Q1 (7.13-21.67) | 1.00 (reference) | 1.00 (reference) | 1.00 (reference) |  |
| Q2 (21.68-28.77) | 3.33 (1.22-9.11) | 5.82 (1.30-26.00) | 1.57 (0.34-7.19) |  |
| Q3 (28.78-39.26) | 5.20 (1.95-13.86) | 8.88 (2.03-38.93) | 2.63 (0.63-11.01) |  |
| Q4 (39.27-675.69) | 7.49 (2.72-20.63) | 11.42 (2.48-52.60) | 5.12 (1.24-21.16) |  |
| P for trend | <0.001 | <0.001 | 0.01 |  |
| Abbreviations: CI, confidence interval; MCI, mild cognitive impairment; Aβ42/40, amyloid beta 42/40 ratio; eGFR, estimated glomerular filtration rate.   1. Adjusted for age, sex, low education, hypertension, diabetes, eGFR, body mass index, history of stroke, smoking habits, sedentariness, and APOE-ε4. | | | | |

| **Supplementary table 4. Multivariable-adjusted risk of all-cause dementia and its subtypes according to plasma quartile levels of each biomarker after excluding participants with MMSE <24, 2012–2017** | | | | |
| --- | --- | --- | --- | --- |
| **Plasma biomarker levels** | **Hazard ratio (95% CI) ^a)^** | | |  |
|  | **All-cause dementia** | **Alzheimer’s disease** | **Non-Alzheimer’s dementia** |  |
| **Aβ42/40** |  |  |  |  |
| Q4 (0.0736-0.2172) | 1.00 (reference) | 1.00 (reference) | 1.00 (reference) |  |
| Q3 (0.0652-0.0735) | 1.00 (0.52-1.93) | 0.73 (0.31-1.71) | 1.69 (0.57-5.03) |  |
| Q2 (0.0595-0.0651) | 1.52 (0.85-2.73) | 1.35 (0.66-2.73) | 2.13 (0.75-6.06) |  |
| Q1 (0.0068-0.0594) | 2.13 (1.22-3.70) | 2.25 (1.17-4.35) | 1.91 (0.67-5.43) |  |
| P for trend | 0.002 | 0.003 | 0.21 |  |
|  |  |  |  |  |
| **Phosphorylated tau181 (pg/mL)** |  |  |  |  |
| Q1 (0.381-3.198) | 1.00 (reference) | 1.00 (reference) | 1.00 (reference) |  |
| Q2 (3.199-4.125) | 0.92 (0.49-1.73) | 1.37 (0.61-3.07) | 0.46 (0.15-1.37) |  |
| Q3 (4.126-5.465) | 0.85 (0.46-1.58) | 1.14 (0.50-2.57) | 0.53 (0.19-1.46) |  |
| Q4 (5.466-44.946) | 1.36 (0.77-2.42) | 2.09 (0.98-4.45) | 0.57 (0.22-1.53) |  |
| P for trend | 0.23 | 0.05 | 0.36 |  |
|  |  |  |  |  |
| **Glial fibrillary acid protein (pg/mL)** |  |  |  |  |
| Q1 (29.72-126.53) | 1.00 (reference) | 1.00 (reference) | 1.00 (reference) |  |
| Q2 (126.54-165.62) | 1.98 (0.85-4.63) | 1.81 (0.61-5.33) | 2.46 (0.62-9.73) |  |
| Q3 (165.63-227.04) | 2.31 (1.003-5.30) | 2.74 (0.99-7.59) | 1.45 (0.32-6.58) |  |
| Q4 (227.05-1095.51) | 5.58 (2.47-12.59) | 5.23 (1.90-14.41) | 6.40 (1.61-25.52) |  |
| P for trend | <0.001 | <0.001 | 0.005 |  |
|  |  |  |  |  |
| **Neurofilament light chain (pg/mL)** |  |  |  |  |
| Q1 (7.13-21.67) | 1.00 (reference) | 1.00 (reference) | 1.00 (reference) |  |
| Q2 (21.68-28.77) | 3.14 (1.15-8.57) | 3.14 (0.87-11.33) | 3.26 (0.65-16.41) |  |
| Q3 (28.78-39.26) | 5.29 (2.00-13.97) | 6.35 (1.86-21.70) | 3.34 (0.65-17.11) |  |
| Q4 (39.27-675.69) | 6.84 (2.50-18.76) | 6.98 (1.92-25.43) | 6.49 (1.28-32.83) |  |
| P for trend | <0.001 | <0.001 | 0.02 |  |
| Abbreviations: CI, confidence interval; MMSE, Mini-Mental State Examination; Aβ42/40, amyloid beta 42/40 ratio; eGFR, estimated glomerular filtration rate.   1. Adjusted for age, sex, low education, hypertension, diabetes, eGFR, body mass index, history of stroke, smoking habits, sedentariness, and APOE-ε4. | | | | |

| **Supplementary table 5. Multivariable-adjusted odds ratio for a decrease of 5 or more points in the MMSE score from 2012 to 2017 according to plasma quartile levels of each biomarker, 2012–2017** | |
| --- | --- |
| **Plasma biomarker levels** | **Odds ratio for a decrease of 5 or more points in the MMSE score from 2012 to 2017 (95% CI) ^a)^** |
| **Aβ42/40** |  |
| Q4 (0.0735-0.2178) | 1.00 (reference) |
| Q3 (0.0652-0.0734) | 0.96 (0.49-1.89) |
| Q2 (0.0570-0.0651) | 1.25 (0.66-2.36) |
| Q1 (0.0067-0.0569) | 2.01 (1.10-3.70) |
|  |  |
| **Phosphorylated tau181 (pg/mL)** |  |
| Q1 (0.381-3.154) | 1.00 (reference) |
| Q2 (3.153-4.074) | 1.23 (0.63-2.39) |
| Q3 (4.073-5.372) | 0.73 (0.36-1.47) |
| Q4 (5.373-42.903) | 1.48 (0.78-2.81) |
|  |  |
| **Glial fibrillary acid protein (pg/mL)** |  |
| Q1 (29.72-124.67) | 1.00 (reference) |
| Q2 (124.68-160.93) | 0.87 (0.40-1.91) |
| Q3 (160.94-220.45) | 1.14 (0.53-2.41) |
| Q4 (220.46-1015.92) | 2.15 (1.02-4.53) |
|  |  |
| **Neurofilament light chain (pg/mL)** |  |
| Q1 (7.26-21.17) | 1.00 (reference) |
| Q2 (21.18-28.00) | 1.35 (0.59-3.07) |
| Q3 (28.01-37.36) | 1.96 (0.88-4.33) |
| Q4 (37.37-675.69) | 2.65 (1.13-6.21) |
| Abbreviations: CI, confidence interval; MMSE, Mini-Mental State Examination; Aβ42/40, amyloid beta 42/40 ratio; eGFR, estimated glomerular filtration rate.   1. Adjusted for age, sex, low education, hypertension, diabetes, eGFR, body mass index, history of stroke, smoking habits, sedentariness, and APOE-ε4. | |

| **Supplementary table 6. C-statistics of each plasma biomarker for developing dementia and the multivariable-adjusted risk for incident dementia in participants with plasma level below cutoff value for plasma amyloid beta 42/40 ratio or above cutoff level for phosphorylated tau181, glial fibrillary acid protein, or neurofilament light chain, 2012-2017** | | | | | | |
| --- | --- | --- | --- | --- | --- | --- |
| ***Plasma biomarker*** | C-statistics  (95%CI) | Cutoff value determined at the point curve that comes closest to the (0.1) coordinate in the ROC curve |  | Multivariable-adjusted risk for incident dementia | | |
|  |  |  |  | Variable | (reference) | Hazard ratio (95% confidence interval) ^a)^ |
| Aβ42/40 | 0.592 (0.542-0.641) | 0.0591 |  | Aβ42/40 ≤0.0591 | (vs. >0.0591) | 2.22 (1.57-3.12) |
| p-tau181 | 0.593 (0.544-0.642) | 4.625 pg/mL |  | p-tau181 ≥4.625 | (vs. <4.625) | 1.35 (0.95-1.92) |
| GFAP | 0.709 (0.666-0.752) | 186.60 pg/mL |  | GFAP ≥186.60 | (vs. <186.60) | 2.56 (1.70-3.85) |
| NfL | 0.699 (0.659-0.741) | 27.10 pg/mL |  | NfL ≥27.10 | (vs. <27.10) | 3.08 (1.89-5.05) |
| Abbreviations: CI, confidence interval; Aβ42/40, amyloid beta 42/40 ratio; p-tau181, phosphorylated tau181; GFAP, glial fibrillary acid protein; NfL, neurofilament light chain; ROC, receiver operatorating characteristic; eGFR, estimated glomerular filtration rate.   1. Adjusted for age, sex, low education, hypertension, diabetes, eGFR, body mass index, history of stroke, smoking habits, sedentariness, and APOE-ε4. | | | | | | |

| **Supplementary table 7. Changes in the risk assessment ability for developing dementia by adding each plasma biomarker divided into binary variables at the cutoff values shown in supplementary table 6 to the predicted dementia risk calculated by the risk score consisting of known risk factors for dementia, 2012–2017** | | | | | | |
| --- | --- | --- | --- | --- | --- | --- |
|  | C-statistics  (95%CI) | P value for difference of C-statistics | Continuous NRI  (95% CI) | P value for NRI | IDI  (95% CI) |  |
| Predicted dementia risk | 0.727 (0.689-0.765) |  |  |  |  |  |
| Predicted dementia risk  + Aβ42/40 ≤0.0591 (vs. >0.0591) | 0.752 (0.715-0.790) | 0.051 | 0.379 (0.212-0.547) | <0.001 | 0.019 (0.009-0.029) | 0.0004 |
| Predicted dementia risk  + pTau181 ≥4.625 pg/mL (vs. <4.625) | 0.734 (0.696-0.771) | 0.30 | 0.333 (0.165-0.502) | 0.01 | 0.002 (-0.0016-0.006) | 0.26 |
| Predicted dementia risk  + GFAP ≥186.60 pg/mL (vs. <186.60) | 0.755 (0.719-0.792) | 0.04 | 0.693 (0.536-0.849) | <0.001 | 0.022 (0.013-0.031) | <0.001 |
| Predicted dementia risk  + NfL ≥27.10 pg/mL (vs. <27.10) | 0.746 (0.709-0.784) | 0.09 | 0.560 (0.418-0.702) | <0.001 | 0.016 (0.011-0.022) | <0.001 |
| Predicted dementia risk  + Aβ42/40 ≤0.0591 (vs. >0.0591)  + GFAP ≥186.60 pg/mL (vs. <186.60) | 0.770 (0.733-0.807) | 0.006 | 0.371 (0.207-0.535) | <0.001 | 0.039 (0.025-0.054) | <0.001 |
| Predicted dementia risk  + Aβ42/40 ≤0.0591 (vs. >0.0591)  + GFAP ≥186.60 pg/mL (vs. <186.60) + NfL≥27.10 pg/mL (vs. <27.10) | 0.777 (0.741-0.814) | 0.002 | 0.643 (0.484-0.802) | <0.001 | 0.048 (0.033-0.063) | <0.001 |
| Predicted dementia risk  + Aβ42/40 ≤0.0591 (vs. >0.0591)  + GFAP ≥186.60 pg/mL (vs. <186.60)  + NfL≥27.10 pg/mL (vs. <27.10)  + p-tau181 ≥4.645 pg/mL (vs. <4.645) | 0.777 (0.741-0.814) | 0.002 | 0.612 (0.451-0.773) | <0.001 | 0.048 (0.033-0.063) | <0.001 |
| Abbreviations: CI, confidence interval; Aβ42/40, amyloid beta 42/40 ratio; p-tau181, phosphorylated tau181; GFAP, glial fibrillary acid protein; NfL, neurofilament light chain  The predicted dementia risk was calculated based on the previously reported risk assessment score consisting of the following variables-namely, age, sex, low education, hypertension, diabetes mellitus, body mass index, history of stroke, current smoking, and sedentariness (reference 35). | | | | | | |
