# Supplementary material for: Plasma biomarkers for predicting the development of dementia in a community‐dwelling older Japanese population
Source: Psychiatry Clin Neurosci. 2024 Apr 12;78(6):362–71. doi: 10.1111/pcn.13661 (PMC11488610; doi:10.1111/pcn.13661)
Supplement: Supplementary file 3 — Data S1: Supplementary Data. [file PCN-78-362-s002.docx]

**Title: Plasma biomarkers for predicting the development of dementia in a community-dwelling older Japanese population**

**Authors:**

Tomoyuki Ohara, M.D., Ph.D.^1,2^, Harutsugu Tatebe, Ph.D.^3^, Jun Hata, M.D., Ph.D.^2,4,5^, Takanori Honda, Ph.D.^2,5^, Mao Shibata, M.D., Ph.D.^2,5,6^, Sayo Matsuura^3^, Tatsuya Mikami, M.D., Ph.D.^7^, Tetsuya Maeda, M.D., Ph.D.^8^, Kenjiro Ono, M.D., Ph.D.^9^, Masaru Mimura, M.D., Ph.D.^10^, Kenji Nakashima, M.D., Ph.D.^11^, Jun-ichi Iga, M.D., Ph.D.^12^, Minoru Takebayashi, M.D., Ph.D.^13^, Takahiko Tokuda, M.D., Ph.D.^3^, and Toshiharu Ninomiya, M.D., Ph.D.^2,5^,

on behalf of the Japan Prospective Studies Collaboration for Aging and Dementia (JPSC-AD) Study Group*

(*The members of the study group are listed in the Acknowledgement section.)

**Affiliations:**

1. Department of Neuropsychiatry, Graduate School of Medical Sciences, Kyushu University, Fukuoka, Japan.

2. Department of Epidemiology and Public Health, Graduate School of Medical Sciences, Kyushu University, Fukuoka, Japan.

3. Department of Functional Brain Imaging, Institute for Quantum Medical Science,

National Institutes for Quantum Science and Technology, Chiba, Japan.

4. Department of Medicine and Clinical Science, Graduate School of Medical Sciences, Kyushu University, Fukuoka, Japan.

5. Center for Cohort Studies, Graduate School of Medical Sciences, Kyushu University, Fukuoka, Japan.

6. Department of Psychosomatic Medicine, Graduate School of Medical Sciences, Kyushu University, Fukuoka, Japan.

7. Department of Preemptive Medicine, Graduate School of Medicine, Hirosaki University, Hirosaki, Japan.

8. Division of Neurology and Gerontology, Department of Internal Medicine, School of Medicine, Iwate Medical University, Iwate, Japan.

9. Department of Neurology, Kanazawa University Graduate School of Medical Sciences, Kanazawa University, Kanazawa, Japan.

10. Department of Neuropsychiatry, Keio University School of Medicine, Tokyo, Japan.

11. National Hospital Organization, Matsue Medical Center, Shimane, Japan.

12. Department of Neuropsychiatry, Ehime University Graduate School of Medicine, Ehime University, Ehime, Japan.

13. Faculty of Life Sciences, Department of Neuropsychiatry, Kumamoto University, Kumamoto, Japan.

**Corresponding Author:**

Toshiharu Ninomiya, M.D., Ph.D.

E-mail adress: t.ninomiya.a47@m.kyushu-u.ac.jp

Department of Epidemiology and Public Health, Graduate School of Medical Sciences, Kyushu University

3-1-1 Maidashi, Higashi-ku, Fukuoka 812-8582, Japan

Tel: (+81) 92-642-6151

Fax: (+81) 92-642-4854

**Acknowledgment**

The authors thank the residents of the town of Hisayama for their participation in the present examination. We are also grateful to the staff members of the Division of Health of Hisayama for their cooperation with the present work. We gratefully acknowledge the diligent work and contributions of all researchers and investigators in the Japan Prospective Studies Collaboration for Aging and Dementia Study Group. Finally, we are grateful for the use of computer resources offered under the category of General Projects by the Research Institute for Information Technology, Kyushu University, which we used to conduct the statistical analyses.

(Participating institutes and principal collaborators in the JPSC-AD Study group)

Kyushu University ([Epidemiology and Public Health] Toshiharu Ninomiya, Jun Hata, and Mao Shibata, Takanori Honda, [Neuropsychiatry] Tomoyuki Ohara, [Ocular Pathology and Imaging Science] Masato Akiyama); Hirosaki University (Shigeyuki Nakaji, Koichi Murashita, Tatsuya Mikami, Songee Jung, and Mina Misawa); Iwate Medical University (Tetsuya Maeda, Naoki Ishizuka, and Hiroshi Akasaka); Shonan Keiiku Hospital (Yasuo Terayama); Japanese Red Cross Morioka Hospital (Hisashi Yonezawa); Kitakami Saiseikai Hospital (Junko Takahashi); Kanazawa University (Kenjiro Ono, Moeko Noguchi-Shinohara, Kazuo Iwasa, and Sohshi Yuki-Nozaki); Kudanzaka Hospital (Masahito Yamada); Keio University School of Medicine (Masaru Mimura, Shogyoku Bun, Hidehito Niimura, Ryo Shikimoto, and Hisashi Kida); Matsue Medical Center (Kenji Nakashima, Yasuyo Fukada, Hisanori Kowa, and Toshiya Nakano); Kawasaki Medical School (Kenji Wada); Tottori Red Cross Hospital (Masafumi Kishi); Ehime University (Tomoki Ozaki, Ayumi Tachibana, Yuta Yoshino, Jun-ichi Iga and Shu-ichi Ueno); Kumamoto University (Minoru Takebayashi, Seiji Yuki, Naoto Kajitani, Yusuke Miyagawa and Shuken Boku); Kindai University Faculty of Medicine (Mamoru Hashimoto); Osaka University Medical School (Manabu Ikeda); National Cerebral and Cardiovascular Center (Yoshihiro Kokubo); Nakamura-Gakuen University (Kazuhiro Uchida and Midori Esaki); Tohoku University (Yasuyuki Taki, Yasuko Tatewaki, and Benjamin Thyreau); University of the Ryukyu (Koji Yonemoto); Osaka Metropolitan University Graduate School of Medicine (Hisako Yoshida); University of Tokyo (Kaori Muto, Yusuke Inoue, and Izen Ri); RIKEN Center for Integrative Medical Sciences (Yukihide Momozawa and Chikashi Terao); Hisayama Research Institute for Lifestyle Diseases (Michiaki Kubo and Yutaka Kiyohara).
